# Antibiotic-Induced Pathobiont Dissemination Accelerates Mortality in Severe Experimental Pancreatitis

**DOI:** 10.3389/fimmu.2017.01890

**Published:** 2017-12-22

**Authors:** Fernanda S. Soares, Flávia C. Amaral, Natália L. C. Silva, Matheus R. Valente, Lorena K. R. Santos, Lívia H. Yamashiro, Mara C. Scheffer, Fernanda V. E. S. Castanheira, Raphael G. Ferreira, Laura Gehrke, José C. Alves-Filho, Luciano P. Silva, André Báfica, Fernando Spiller

**Affiliations:** ^1^Laboratory of Immunobiology, Federal University of Santa Catarina (UFSC), Florianópolis, Brazil; ^2^Microbiology Laboratory, University Hospital, Federal University of Santa Catarina (UFSC), Florianópolis, Brazil; ^3^Department of Pharmacology, Ribeirao Preto Medical School, University of Sao Paulo, Ribeirao Preto, Brazil; ^4^Embrapa Genetic Resources and Biotechnology, Brasilia, Brazil; ^5^Institute of Biological Sciences, University of Brasilia, Brasilia, Brazil; ^6^Department of Microbiology, Immunology and Parasitology, Federal University of Santa Catarina (UFSC), Florianópolis, Brazil; ^7^Department of Pharmacology, Federal University of Santa Catarina (UFSC), Florianópolis, Brazil

**Keywords:** experimental acute pancreatitis, meropenem-induced pathobiont, *Enterococcus gallinarum*, microbiota, sepsis, antibiotics

## Abstract

Although antibiotic-induced dysbiosis has been demonstrated to exacerbate intestinal inflammation, it has been suggested that antibiotic prophylaxis may be beneficial in certain clinical conditions such as acute pancreatitis (AP). However, whether broad-spectrum antibiotics, such as meropenem, influence the dissemination of multidrug-resistant (MDR) bacteria during severe AP has not been addressed. In the currently study, a mouse model of obstructive severe AP was employed to investigate the effects of pretreatment with meropenem on bacteria spreading and disease outcome. As expected, animals subjected to biliopancreatic duct obstruction developed severe AP. Surprisingly, pretreatment with meropenem accelerated the mortality of AP mice (survival median of 2 days) when compared to saline-pretreated AP mice (survival median of 7 days). Early mortality was associated with the translocation of MDR strains, mainly *Enterococcus gallinarum* into the blood stream. Induction of AP in mice with guts that were enriched with *E. gallinarum* recapitulated the increased mortality rate observed in the meropenem-pretreated AP mice. Furthermore, naïve mice challenged with a mouse or a clinical strain of *E. gallinarum* succumbed to infection through a mechanism involving toll-like receptor-2. These results confirm that broad-spectrum antibiotics may lead to indirect detrimental effects during inflammatory disease and reveal an intestinal pathobiont that is associated with the meropenem pretreatment during obstructive AP in mice.

## Introduction

Gut microbiota have emerged as an important hub in the regulation of the inflammatory response (1). Accordingly, a number of conditions, such as rheumatoid arthritis, diabetes, and inflammatory bowel disease, have been associated with gut dysbiosis (2). Although it is difficult to distinguish whether dysbiosis is the cause or consequence of inflammation, a body of evidence has suggested that the use of antibiotics can cause dysbiosis, leading to the dissemination of microbiota-derived multidrug-resistant (MDR) bacteria and poor inflammatory disease outcomes (3–5). For example, using a mouse model of intestinal inflammation induced by dextran sodium sulfate, Ayres and co-workers demonstrated that an antibiotic treatment lead to gut proliferation and systemic spreading of an MDR *Escherichia coli* strain, resulting in the rapid death of mice (3). In another study, Knoop et al. (5) demonstrated that a single dose of antibiotics induces dysbiosis-mediated bacterial translocation and worsens the dextran sodium sulfate-induced inflammatory response. These two examples indicate that certain antibiotics can play a detrimental role in intestinal inflammation. However, whether antibiotic-induced dysbiosis plays a role in the severity of other life-threatening inflammatory conditions, such as acute pancreatitis (AP), is less understood.

Acute pancreatitis is a systemic inflammatory response that is predominantly caused by obstruction of the pancreatic duct by gallstones and has a worldwide incidence of approximately 15 cases per 100,000 individuals per year (6). Twenty percent of AP patients will develop severe necrotic AP, and the combination of multiple organ failure and infection of the necrotic pancreas leads to mortality in up to 43% of patients (7). Therefore, a prophylactic antibiotic management regimen has been proposed to prevent infected pancreatic necrosis in the 1990s (8). The broad-spectrum carbapenems, such meropenem, are frequently used in the prophylaxis of AP patients because of their higher penetration of pancreatic tissue compared to other antibiotics (9–13). Carbapenems are also frequently used in septic patients as an empiric treatment prior to bacterial identification (14). Nowadays, guidelines for the management of AP do not recommend antibiotic prophylaxis in AP (15). However, this information is not precisely followed in clinical practice since antibiotic prophylaxis is still worldwide used in a fraction of AP patients (16–20). The benefits of antibiotic prophylaxis for patients with severe AP remain controversial. For instance, Dellinger et al. showed that prophylaxis treatment with meropenem did not significantly decrease pancreatic or peripancreatic infection, mortality, or the requirement for surgical intervention (21). In contrast, Rokke et al. demonstrated that a prophylactic treatment with a related drug, imipenem, decreased the rates of septic complications without affecting mortality or the need for surgical intervention (22). In a meta-analysis that included 11 studies with a total of 864 patients, Lim et al. found no significant difference in the incidence of infected pancreatic necrosis in patients subjected to antibiotic prophylaxis (23). Although these observations suggest that antibiotic prophylaxis or treatment of AP may be beneficial in some but not all cases, whether antibiotics influence the disease severity and spread of MDR pathobionts remains to be investigated. To date, only a few studies have evaluated the effects of meropenem on the outcome of experimental AP (24, 25) and the identities of the resistant strains that are selected by meropenem in patients or in experimental AP have not been addressed.

In the present study, an established biliopancreatic duct obstruction (BDO) model of AP was employed (26) to study whether meropenem pretreatment influences bacterial dissemination and disease outcome during severe AP. The results presented here show that meropenem pretreatment induces dissemination of gut MDR bacterial species, mainly *Enterococcus gallinarum*, which is associated with an accelerated mortality of AP mice. Furthermore, the mortality induced by *E. gallinarum* involves a TLR2-dependent pathway. These data reveal *E. gallinarum* as a pathobiont in severe experimental AP that emerges during meropenem pretreatment.

## Materials and Methods

### Mice

Care and treatment of the animals were based on the *Guide for the Care and Use of Laboratory Animals* (27). Specific-pathogen-free (SPF) male and female, Balb/C, C57BL/6 wild-type (Wt), and C57BL/6-TLR2-deficient (TLR2^−/−^) (8–10 weeks old) mice were housed under SPF conditions at the Animal Facility of the Department of Microbiology, Immunology and Parasitology from Federal University of Santa Catarina (UFSC). A total of 641 mice were used in this study. C57BL/6 Wt and Balb/C mice were purchased from Jackson Laboratory. TLR2^−/−^ mice generated by Dr. Shizuo Akira (Osaka University, Japan) (28) were a kind gift from Ricardo T. Gazzinelli (Federal University of Minas Gerais, Brazil). Mice were housed in cages at 21 ± 2°C with free access to water and food. All animal experiments were approved by the Animal Welfare Committee of the UFSC (PP00880).

### AP Model

Acute pancreatitis was induced by BDO, as described (26). Briefly, mice were anesthetized with xylazine (2 mg/kg, i.p.) followed by isoflurane (3–5 vol%). A 1-cm midline incision was made in the anterior abdomen, and the biliopancreatic duct was exposed at its orifice in the duodenal wall. The duct was dissected, and a tourniquet consisting of a cotton sewing and 2 mm of a P10 cannula was looped around the biliopancreatic duct adjacent to the duodenal wall. In the sham-operated (Sham) animals, the biliopancreatic duct was dissected but not obstructed. All animals received 1 mL of saline subcutaneously immediately after surgery. The obstructive model employed in the present study mimics the mechanical obstruction of the bile and pancreatitic ducts induced by biliar gallstones in AP patients, one of the main etiology of AP (6). The obstructive model of AP leads to disease development by self-mediators, which is a better representation of the natural course of the disease when compared to other models of severe AP, when chemical agents are intraductally administrated (24, 25).

### Determination of Serum Biochemical Markers

Colorimetric assay kits were used to determine the serum activity of alanine aminotransferase, aspartate aminotransferase, amylase, lipase, alkaline phosphatase, and lactate dehydrogenase and the serum concentrations of bilirubin, urea, and creatinine (Labtest Diagnóstica, Brazil).

### Meropenem Treatments

Mice were pretreated or treated intraperitoneally (i.p.) with saline or meropenem (Eurofarma, SP, Brazil, 100 mg/kg, 12/12 h) for 3 days and then subjected to surgery. The surgical procedure was performed 12 h after the last dose in the pretreated groups, and posttreatment started 12 h after surgery. Mice were also pretreated with meropenem for 3 or 5 days (30 or 100 mg/kg, 12/12 h) to evaluate the tissue injury induced by the antibiotic treatment.

### Clindamycin/Ceftriaxone Treatment

Mice were pretreated i.p. (12/12 h) with saline or clindamycin (Hipolabor, MG, Brazil, 25 mg/kg) plus ceftriaxone (União Química, SP, Brazil, 30 mg/kg) for 3 days and then subjected to surgery.

### Microbiota Enrichment

To reduce the intestinal bacterial community, mice were treated with a cocktail of broad-spectrum antibiotics, as described previously (29). Animals were first exposed to amphotericin-B (Sigma-Aldrich, St. Louis, MO, USA, 1 mg/kg body weight, BW) delivered by gavage during 3 days, 12/12 h. From days 3 to 5, ampicillin (Sigma-Aldrich, 1 mg/mL) was added into the drinking water, and the mice were gavaged 12/12 h with a cocktail of antibiotics consisting of vancomycin (Sigma-Aldrich, 50 mg/kg BW), neomycin (Sigma-Aldrich, 100 mg/kg BW), metronidazole (Sigma-Aldrich, 100 mg/kg BW), and amphotericin-B (1 mg/kg BW). Additionally, from days 3 to 15, mice were i.p.-treated daily, with ciprofloxacin (Sigma-Aldrich, 50 mg/kg BW). From days 16 to 27, mice were gavaged daily with 100 µL (100 mg/mL) of a feces suspension or with 100 µL (1 × 10^8^ colony-forming units, CFUs) of an *E. gallinarum* suspension. Fresh feces were collected from naïve mice, homogenized in sterile PBS and decanted for 30 min at RT. Animals were then exposed to supernatants of the feces suspension. *E. gallinarum* was cultivated in brain–heart infusion liquid medium at 37°C until an optical density of 0.60 at 600 nm was reached, and the bacteria were washed three times with PBS prior to animal administration. On day 28, mice were subjected to Sham or AP induction.

### Bacterial Counts

The bacterial count was determined as previously described (30). Briefly, samples were collected under sterile conditions, and 10 µL of serial dilutions were seeded on agar dishes containing Muller-Hinton, blood agar Muller-Hinton, or bile esculin azide agar with 6 µg/mL of vancomycin (Difco Laboratories, Detroit, MI, USA) and grown at 37°C aerobically for 24 h. The lumina of the intestinal samples were washed with PBS for intestinal CFU quantification. The results were expressed as the mean ± SEM log of CFU per milliliter, grams of feces, or milligrams of tissue.

### Bacterial Identification

We identified bacterial species using biochemical and microbiological methods, as described (31) and using the Vitek2 automated system (bioMérieux, see Tables S1–S8 in Supplementary Material for details regarding the bacterial identification).

### Colony PCR

Individual colonies of *E. coli* and *E. gallinarum* were resuspended in sterile PBS and boiled for 10 min. These samples were used for PCR analysis with the following primers for the *vanC1* gene: F_5′GGTATCAAGGAAACCTC3′ and R_5′CTTCCGCCATCATAGCT3′ (32). PCR was performed on a Mastercycler Gradient™ thermocycler (Eppendorf, Germany) with the following conditions: 95°C (10 min) followed by 35 cycles of 95°C (1 min), annealing at 54°C (30 s), 72°C (33 s), with a final extension step at 72°C (5 min). The concentration of magnesium used was 2.0 mM.

### Antibiotic Susceptibility Assays

*Enterococcus gallinarum* susceptibility to vancomycin, clindamycin, streptomycin, ampicillin, and meropenem was determined using a disk diffusion or Etest assay. *E. gallinarum* was grown in brain–heart infusion liquid medium at 37°C until an optical density of 0.60 at 600 nm was reached, and aliquots (including a 0.5 McFarland standard) of the cultures were spread onto Muller-Hinton agar plates; 6-mm Whatman disks loaded with the appropriate antibiotic were added to the agar. Plates were incubated overnight at 37°C, and the zone of bacterial growth inhibition was measured.

### *E. gallinarum* Infection

*Enterococcus gallinarum* (FMC012174) was isolated from the blood of the AP mice that were pre-exposed to meropenem. The clinical strain of *E. gallinarum* (UH-FMC001) was isolated from the blood of a patient with sepsis and was a gift from the Microbiology Laboratory, University Hospital, UFSC. The *E. gallinarum* strains were grown in brain–heart infusion liquid medium at 37°C until an optical density of 0.60 at 600 nm was reached, and the strains were washed three times and resuspended with PBS. Six hours later, the AP induction mice were intravenously (i.v.) treated with 100 µL of saline, 100 µL of 1 × 10^8^ CFU of live *E. gallinarum* or 100 µL of 1 × 10^8^ CFU of heat-killed *E. gallinarum*. Samples of *E. gallinarum* were boiled for 15 min, and no bacterial growth was observed in these samples on the blood agar dishes (37°C, 24 h). In another experimental set, Wt or TLR2^−/−^ naïve mice were i.p.-treated with 200 µL of 1 × 10^9^ CFU of the live mouse or clinical strains of *E. gallinarum*.

### Histological Examination

Mice were euthanized 24 h after surgery, and the pancreases were fixed by immersion in 5% paraformaldehyde, dehydrated, and embedded in paraffin wax. Three-micron-thick sections were stained with hematoxylin and eosin for histological examination. The scoring criteria for edema, inflammatory cell infiltration, and necrosis were as follows: 0, absent; 1, mild (5–10% of tissue); 2, moderate (10–30% of tissue); and 3, severe (30% of the tissue). The mean ± SEM represented the sum of scores (range from 0 to 9) of the overall damage to the pancreas. Blind histological assessments were performed by a pathologist.

### Leukocyte Migration to Peritoneal Cavity

Leukocyte migration was assessed 1, 4, and 24 h after surgery, as described (33).

### Leukocyte Infiltration in Lung

Lungs were perfused with PBS/EDTA (1 mM) before harvest from mice. Samples were collected 16 h after the last treatment. Lungs were passed through 40-µm nylon cell strainers and single cell suspensions were centrifuged in 35% Percoll^®^ solution (315 mOsm/kg, Sigma-Aldrich) for 15 min at 700 × *g* to enrich leukocytes populations. Pelleted cells were then collected, and erythrocytes were lysed. Single cell suspensions from individual mice were stained for flow cytometry analysis, as described (34). Cells were stained with anti-mouse antibodies (BD Pharmingen) to GR1 (clone RB6-8C5), CD11b (clone M1/70) and F4/80 (clone BM8). Data were collected using a FACSCanto II (BD Immunocytometry Systems) and analyzed using FlowJo software.

### Statistics

Data are reported as the means ± SEM of the values obtained from three independent experiments. The mean values for the different groups were compared by analysis of variance (ANOVA). If significance was determined, individual comparisons were subsequently tested with Bonferroni’s *t-*test for unpaired values. Bacterial counts were analyzed by the Mann–Whitney *U-*test or unpaired *t*-test using a parametric test with Welch’s correction. Survival curves were plotted using the Kaplan–Meier method and then compared using the log-rank method and Gehan-Wilcoxon test. Data were analyzed using GraphPad Prism version 6.00 for Mac (GraphPad Software, USA). A *P* < 0.05 was considered significant.

## Results

### BDO Induces Severe AP

**Figure 1:**
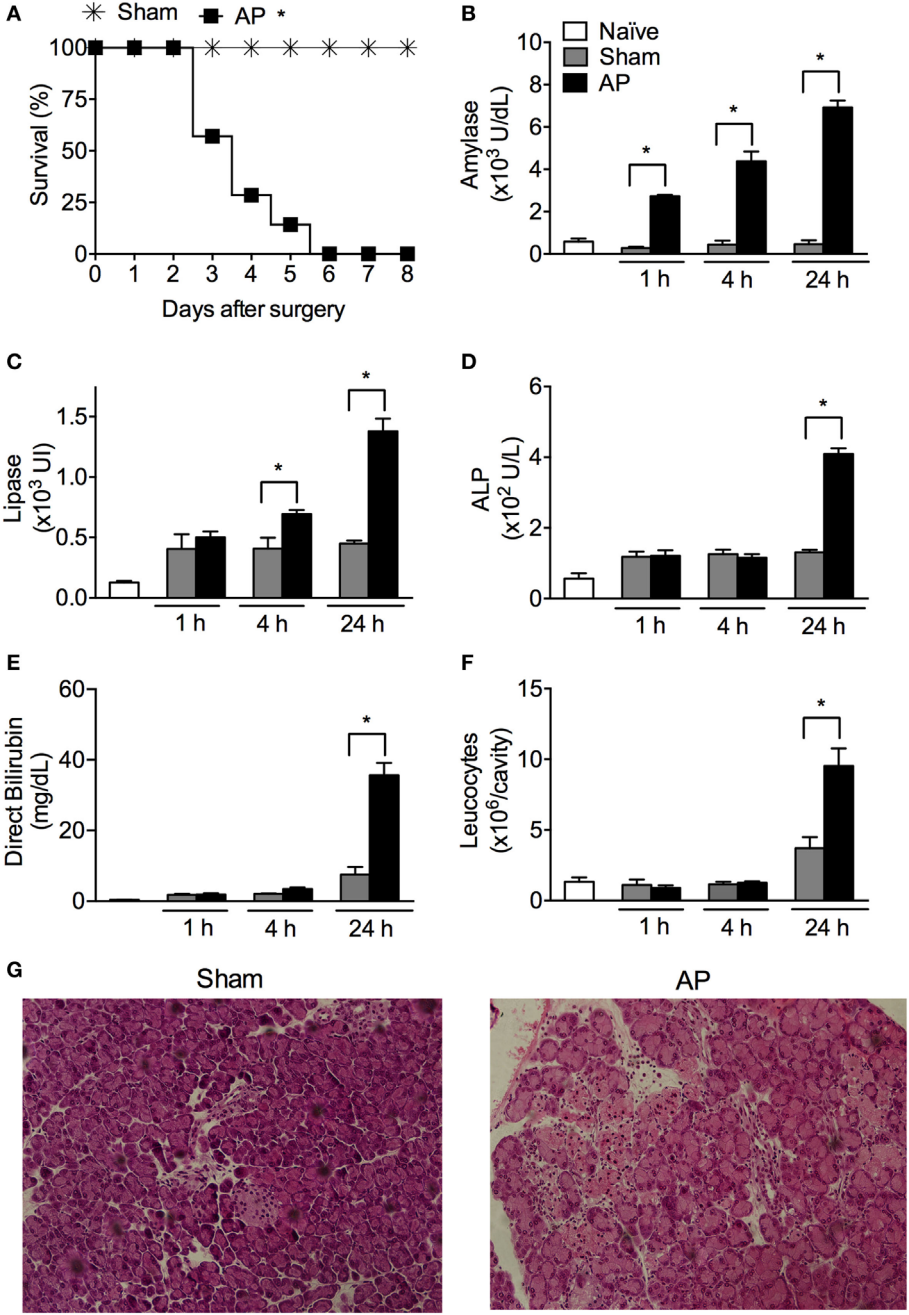
Obstruction of the biliopancreatic duct triggers severe acute pancreatitis (AP) in mice. **(A)** Survival of C57BL/6 mice subjected to AP (*n* = 31) or the sham operation (Sham, *n* = 16). Serum concentrations of amylase **(B)**, lipase **(C)**, alkaline phosphatase (ALP) **(D)**, direct bilirubin **(E)**, and total leukocytes **(F)** in the peritoneal cavity lavage were evaluated 1, 4, and 24 h after surgery in AP (*n* = 48), Sham (*n* = 27), or naïve mice (*n* = 27). **(G)** Histomorphological analysis of pancreas collected from mice 24 h after Sham or AP induction. These experiments were performed independently three times. *P < 0.05 compared with Sham mice.

Gallstone disease is one of the most common etiologies of AP (6). Therefore, we modeled obstructive AP with BDO. All mice subjected to BDO died within 6 days after surgery, whereas all sham-operated (Sham) mice survived (Figure 1A). Compared to Sham animals, BDO animals displayed a significant increase in serum amylase/lipase and alkaline phosphatase/direct bilirubin, which are biomarkers associated with AP and biliary obstruction, respectively (Figures 1B–E). Additionally, we detected a significant augmentation of leukocytes in the peritoneal cavity of the AP mice compared to the Sham mice (Figure 1F). Histological examination of the pancreas indicated that the obstructive experimental AP model resembled major features of severe AP in patients (35). The pancreas from the Sham group showed a mean of histological scores of 1.75 (with a range from 0 to 2) with edema and inflammatory cell infiltration as the predominant characteristics (Figure 1G). Pancreatic tissue sections of the AP group exhibited a mean histological score of 5.0 (with a range from 3 to 7), characterized by diffuse acinar cell necrosis, inflammatory cell infiltration, and edema. These results confirm that the obstructive mouse model employed induces severe AP.

### Meropenem Treatment Accelerates the Mortality of AP Mice

**Figure 2:**
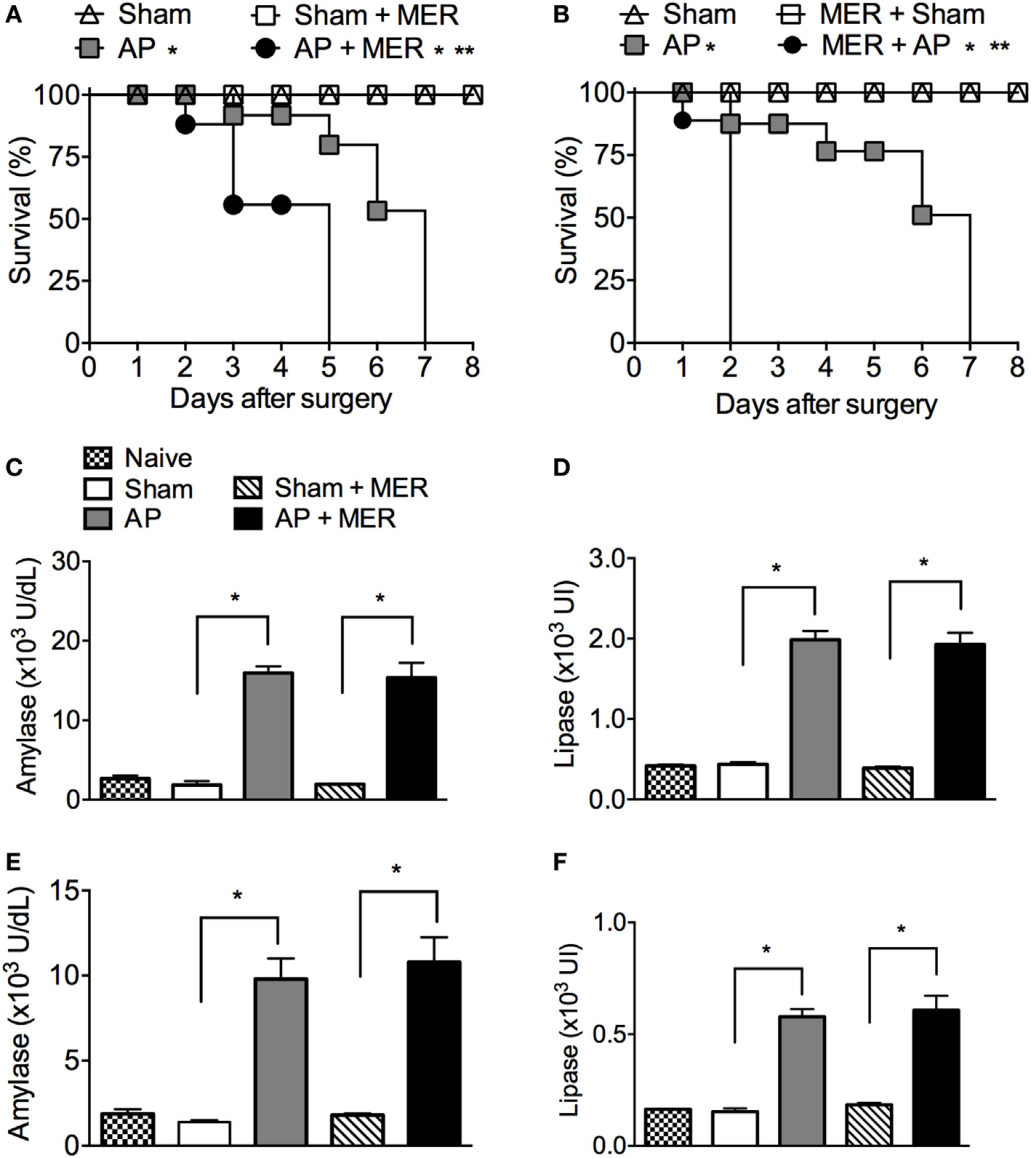
Meropenem accelerates the mortality rate of mice with acute pancreatitis (AP). **(A)** Survival rate of mice subjected to the sham operation (Sham, *n* = 9), Sham mice posttreated with meropenem (Sham + MER, 100 mg/kg, i.p., 12/12 h, 3 days, *n* = 9), AP posttreated with saline (200 µL, i.p., 12/12 h, 3 days, *n* = 15), or AP posttreated with meropenem (AP + MER, 100 mg/kg, i.p., 12/12 h, 3 days, *n* = 25). Posttreatments started 12 h after the surgeries. *P < 0.0001 compared with the Sham groups; **P < 0.0001 compared with the AP mice. **(B)** Survival rate of mice subjected to the procedures described in **(A)**, with a modification that saline and meropenem were given as a pretreatment (100 mg/kg, i.p., 12/12 h, 3 days). Surgeries were performed 12 h after the last dose of saline or meropenem (Sham *n* = 9, Sham + MER *n* = 12, AP *n* = 16, and AP + MER *n* = 31). *P < 0.0001 compared with the Sham groups; **P < 0.0001 compared with the AP mice. These experiments were performed independently three times. Serum concentrations of **(C)** amylase and **(D)** lipase of mice subjected to Sham (*n* = 6), Sham + MER (*n* = 6), AP (*n* = 10), and AP + MER (*n* = 12). Samples were collected 60 h after surgery. Serum concentrations of **(E)** amylase and **(F)** lipase in mice subjected to Sham (*n* = 6), MER + Sham (*n* = 6), AP (*n* = 10), and MER + AP (*n* = 10). Samples were collected 24 h after surgery. *P < 0.0001 compared with the Sham or naïve mice. These experiments were performed independently two times.

As meropenem is a broad-spectrum antibiotic used as a prophylactic treatment in severe AP (36), the effects of meropenem on the outcome of experimental AP were investigated. Posttreatment of AP C57BL/6 mice with meropenem significantly accelerated animal mortality (Figure 2A). As expected, all Sham mice treated with saline or meropenem survived (Figure 2A). To investigate the factor(s) underlying the observed mortality induced by meropenem in AP C57BL/6 mice, animals were pretreated with the antibiotic for 3 days, and AP was induced by BDO 12 h after the last dose. Meropenem-pretreated AP mice displayed a strong reduction in survival rate (median survival of 2 days) compared to saline-pretreated AP animals (median survival of 7 days, Figure 2B). In the two meropenem-exposed AP groups (posttreatment and pretreatment), the serum levels of amylase and lipase were equal to those in the saline-exposed AP mice (Figures 2C–F), suggesting that meropenem did not directly affect AP development.

As reported in previous studies (37, 38), treatment of naïve mice for 3 or 5 days did not increase the serum activities of alanine aminotransferase, aspartate aminotransferase, urea, creatinine, or lactate dehydrogenase (Figures S1A–E in Supplementary Material), indicating the faster mortality observed in the meropenem-treated animals was not due to acute toxicity mediated by this antibiotic. In addition, the effect of antibiotic on immune system activation was evaluated. Leukocyte infiltration in the lungs is a sensitive hallmark of systemic inflammation and a key step to lung injury in AP (39–42). Figure S2 in Supplementary Material shows that antibiotic treatment did not change the numbers of neutrophils (GR1^high^CD11b^+^) and monocytes (F4/80^high^CD11b^high^) in lungs of naïve or Sham mice, however, significantly increased the F4/80^high^CD11b^high^ cells in the lungs of AP mice. These results suggest whether antibiotic induced an activation of the immune system, it is not enough to generate a systemic inflammatory response in naïve or Sham mice. However, in the context of experimental AP, the antibiotic pretreatment could worsen the outcome of systemic inflammation.

**Figure 3:**
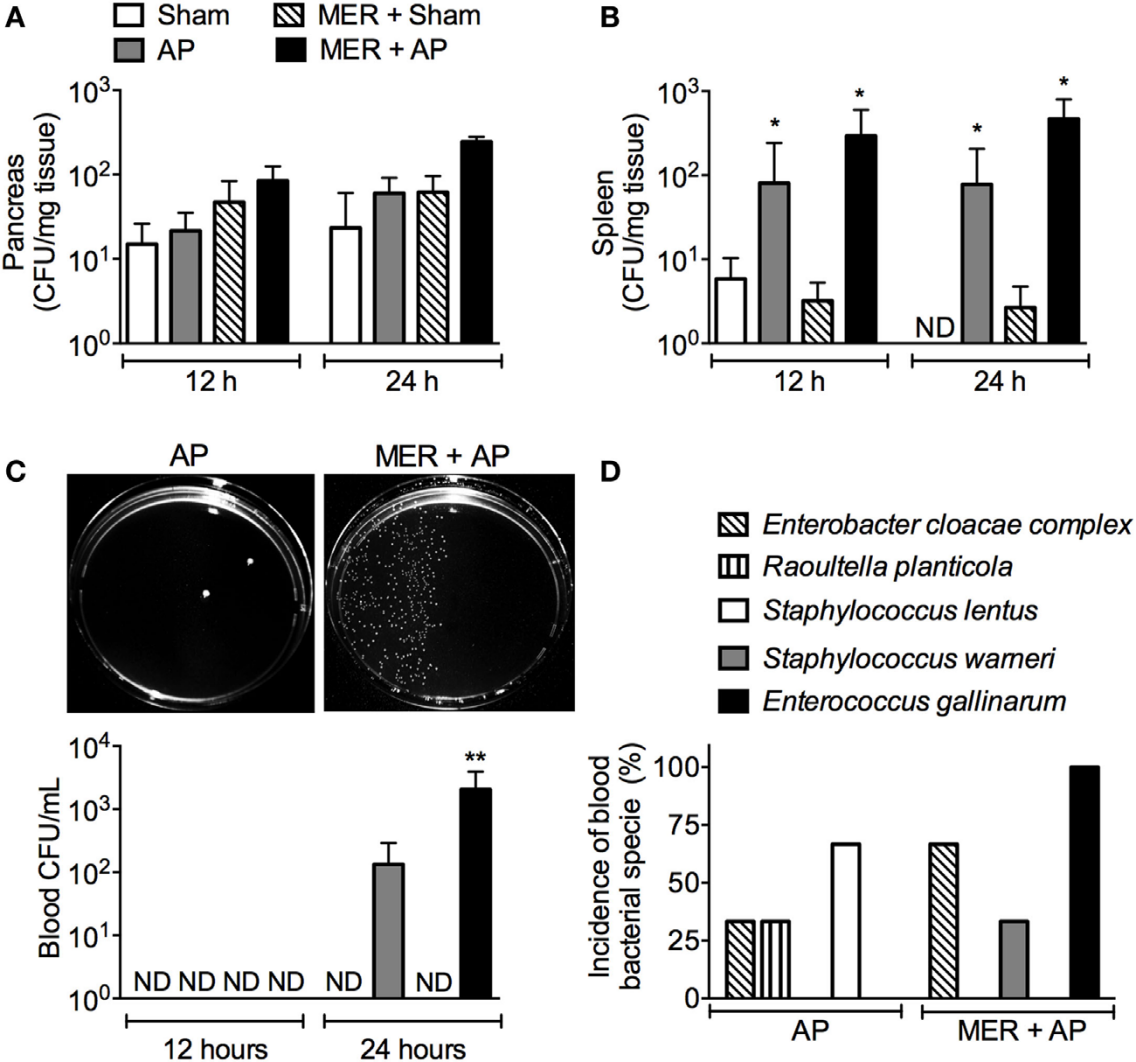
Meropenem pretreatment increases the colony-forming units (CFUs) in the blood of mice with acute pancreatitis (AP). The bacterial load in the **(A)** pancreas, **(B)** spleen, and **(C)** blood was evaluated 12 or 24 h after the surgeries in samples harvested from the pretreated mice. *P < 0.05 compared with the Sham mice. **P < 0.05 compared with the AP mice. Sham (*n* = 9), MER + Sham (*n* = 9), AP (*n* = 15), and MER + AP (*n* = 15). These experiments were performed independently three times. The pictures of the Mueller-Hinton agar plates provided in panel **(C)** show an example of the bacterial load in the blood samples from the AP or meropenem-pretreated AP mice 24 h after the surgeries. The pictures were converted to black and white, and the contrast of the whole picture was increased using the Adobe Photoshop 7.0 software (see original pictures in Figure S6 in Supplementary Material). **(D)** The CFUs that increased in the blood samples from AP or MER + AP were identified using the Vitek2 method. The results were expressed as the incidence of bacterial species in the blood (we analyzed 9 samples from a total of 15 mice). ND, not detectable.

Since antibiotic-induced bacterial dissemination has been associated with a poor inflammatory disease outcome (3, 5), the effect of the meropenem pretreatment on the CFU number in tissues and blood was investigated. The CFU numbers in the pancreas were similar in all groups (Figure 3A). In the spleen, the number of CFUs increased in the AP and meropenem-pretreated AP mice compared with the Sham groups but were similar between the AP and meropenem-pretreated AP mice (Figure 3B). However, 24 h after surgery, a significant increase in the CFU content in the blood was observed in meropenem-pretreated AP mice compared to AP mice (Figure 3C). The pictures provided in Figure 3C show an example of the high CFU content in the blood from meropenem-pretreated AP mice.

Compared to AP mice, meropenem-pretreated AP animals displayed approximately 10 times more CFUs in the small intestine but not in the colon or cecum (Figure S3 in Supplementary Material). Next, automated, classical biochemically based and microbiology-based methods were employed to identify the bacterial species. Approximately 80% of bacteria found in the small intestine were identified as *E. gallinarum* (Table S1 in Supplementary Material). The remaining 20% of the bacteria identified were *Staphylococcus epidermidis, Staphylococcus lentus*, and *Bacillus* spp. (Tables S2, S3, and S8 in Supplementary Material).

Importantly, *E. gallinarum* was also found in high abundance in the blood samples of 100% of meropenem-pretreated AP mice (9/9), indicating bacterial translocation following AP disease (Figure 3D). Although in lower frequency, *Enterobacter cloacae* complex strains (6/9) and *Staphylococcus warneri* (3/9) were observed in the blood of antibiotic-treated AP mice (Figure 3D; Tables S1, S4, and S5 in Supplementary Material). *E. cloacae complex* (3/9), *Raoultella planticola* (3/9), and *S. lentus* (6/9) were identified in saline-pretreated AP mice (Figure 3D; Tables S3, S4, and S6 in Supplementary Material). These results suggest a major change in the bacterial profile of the blood from meropenem-pretreated AP animals and reveal the emergence of *E. gallinarum*.

Since *E. gallinarum* was the most frequent species observed in the blood of meropenem-pretreated AP mice, this result was investigated further. *E. gallinarum* was positive for the VanC1 gene (Figure S4 in Supplementary Material) (43) and showed low resistance to vancomycin (MIC = 6–8 µg/mL), intermediate resistance to meropenem (zone of inhibition = 16 mm), and high resistance to clindamycin, ceftriaxone, and streptomycin (zone of inhibition = 0 mm), but *E. gallinarum* was sensitive to ampicillin (zone of inhibition = 30 mm). Notably, pretreatment (12/12 h, 3 days) of C57BL/6 and Balb/C mice with clindamycin and ceftriaxone also induced major reductions in the survival rates of AP mice (Figure S5 in Supplementary Material).

### *E. gallinarum*-Recolonized Mice Show Increased Disease Severity following AP

**Figure 4:**
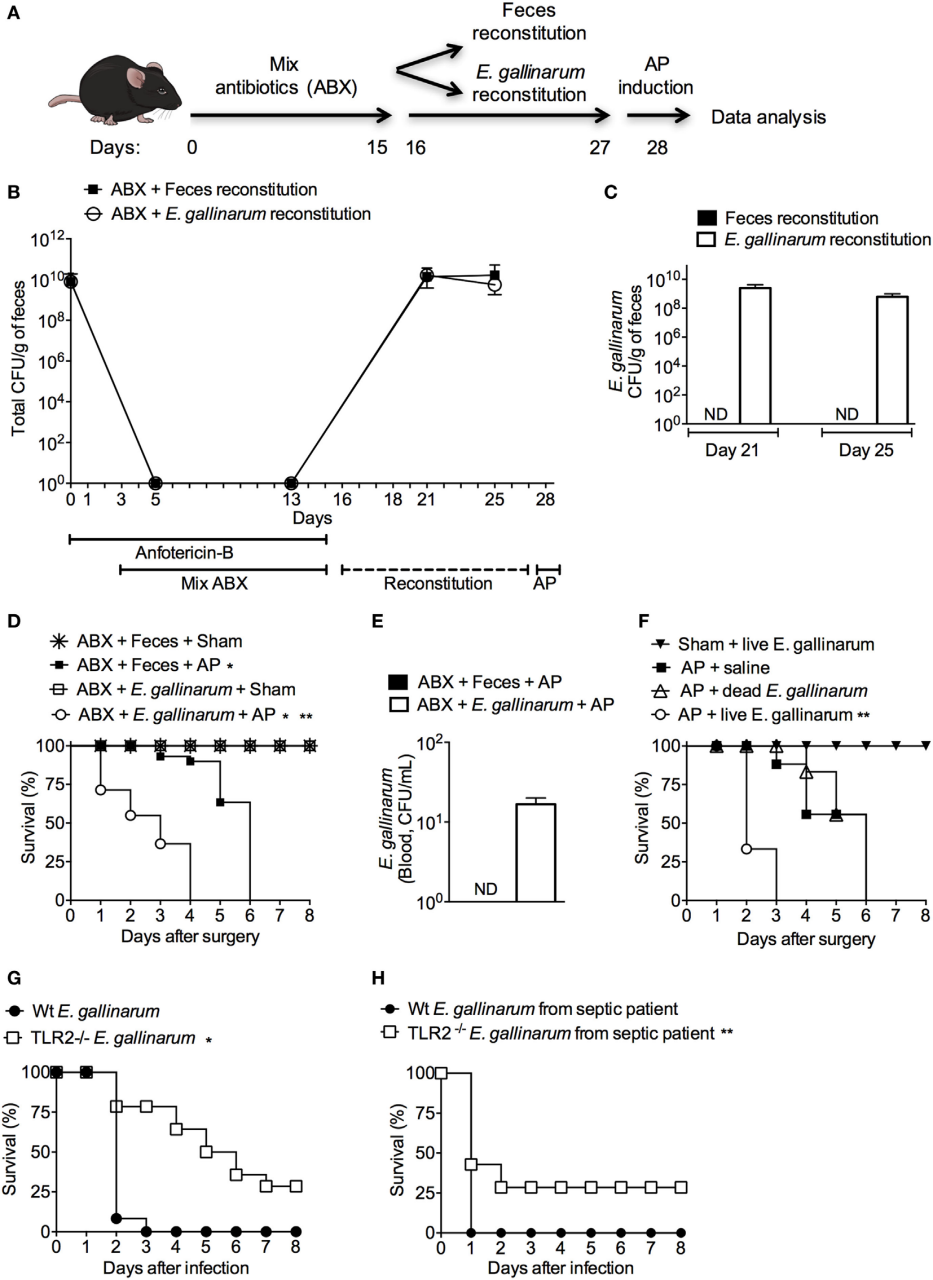
Microbiota enrichment with *Enterococcus gallinarum* induces enhanced mortality of acute pancreatitis (AP) mice. **(A)** Experimental design for the intestinal bacterial community reduction, intestinal bacterial reconstitution, AP induction, and data analysis (mice design from https://mindthegraph.com). **(B)** Fresh fecal samples were collected from mice prior to the experiment (day 0) and spread on Mueller-Hinton agar plates; the results were expressed as colony-forming unit (CFU)/g of feces. Beginning on the following day (day 1) and continuing to day 15, mice were exposed to a mix of antibiotics (ABX). From day 16 to day 27, mice were gavaged daily with 100 µL (100 mg/mL) of the feces suspension or with 100 µL (1 × 10^8^ CFU) of the *E. gallinarum* suspension (see Materials and Methods). Fresh feces were also collected from mice on days 5, 13, 21, and 25 and spread on Mueller-Hinton agar plates. **(C)** Fresh feces collected on days 21 and 25 were spread on bile esculin azide agar plates with 6 µg/mL of vancomycin, and the CFUs that grew on samples from mice treated with the *E. gallinarum* suspension were identified as *E. gallinarum* using the Vitek2 method. The results were expressed as *E. gallinarum* CFU/g of feces. **(D)** The survival rates of the Sham or AP mice that received feces or the *E. gallinarum* suspension (Sham *n* = 9, MER + Sham *n* = 9, AP *n* = 15, and MER + AP *n* = 15); *P < 0.0001 compared with the Sham groups; **P < 0.0001 compared with AP mice. **(E)** Blood samples were collected 24 h after surgery and spread on bile esculin azide agar plates with 6 µg/mL of vancomycin. CFUs grew on the samples from mice treated with the *E. gallinarum* suspension were identified as *E. gallinarum* using the Vitek2 method. The results were expressed as *E. gallinarum* CFU/mL of blood. **(F)** The survival rates of the AP mice treated intravenously (i.v.) with saline, heat-killed (dead, *n* = 15) *E. gallinarum* (1 × 10^8^ CFU/mouse, *n* = 15) or live *E. gallinarum* (1 × 10^8^ CFU/mouse, *n* = 15) and Sham mice treated i.v. with live *E. gallinarum* (1 × 10^8^ CFU/mouse, *n* = 8); *P < 0.0001 compared with AP + dead *E. gallinarum* or AP + saline. The survival rates of C57BL/6 (Wt) and TLR2-deficient (TLR2^−/−^) mice challenged with intraperitoneal administration of **(G)** *E. gallinarum* (1 × 10^9^/mouse, *n* = 15) isolated from mice or **(H)** *E. gallinarum* (1 × 10^9^/mouse, *n* = 15) isolated from a septic patient. *P < 0.0001 compared with Wt *E. gallinarum*. **P < 0.01 compared with Wt *E. gallinarum* from septic patient. These experiments were performed independently three times. ND, not detectable.

To investigate the existence of a possible link between *E. gallinarum* and the severity of AP, the intestinal microbiota of mice were suppressed with a cocktail of broad-spectrum antibiotics (29) and repopulated with a fecal or *E. gallinarum* suspension (Figure 4A). As expected (29), the cocktail of broad-spectrum antibiotics depleted all fecal cultivable aerobic bacteria. Oral administration of fecal or *E. gallinarum* suspensions to antibiotic-treated mice resulted in high levels of intestinal recolonization (Figure 4B). Moreover, fecal samples from mice treated orally with *E. gallinarum*, but not with feces, contained ~10^8^ CFU/g (Figure 4C) in the vancomycin-resistant enterococci (VRE) selective agar medium. All of these colonies were *E. gallinarum* as confirmed using the Vitek2 method. Strikingly, compared to mice recolonized with the fecal suspension, animals exposed to *E. gallinarum* displayed enhanced mortality during AP onset (Figure 4D). This effect was associated with *E. gallinarum* translocation into the blood (Figure 4E). Furthermore, intravenous administration of live *E. gallinarum* in AP mice induced significantly higher mortality rates compared to AP animals administered either saline or dead *E. gallinarum* (Figure 4F). Taken together, these results indicate that *E. gallinarum* is sufficient to exacerbate the severity of AP.

To determine if isolated *E. gallinarum* induces sepsis in naïve mice, Wt animals were challenged with these bacteria, and the survival rates were analyzed. All mice exposed to a systemic challenge with *E. gallinarum* died within 3 days of the infection (Figure 4G). Since *E. gallinarum* could induce cellular activation through a TLR2/6-dependent pathway (44) and since this receptor plays a pivotal role in mortality during severe sepsis (30), we challenged TLR2-deficient mice with *E. gallinarum*. Figure 4G shows that ~30% of TLR2-deficient animals survived after a lethal challenge with *E. gallinarum*. Similarly, TLR2-deficient mice were found to be partially resistant following exposure to a clinical strain of *E. gallinarum* isolated from a septic patient (Figure 4H). These data suggest that TLR2 is involved in the host pathogenesis of the *E. gallinarum* infection.

## Discussion

In the present study, we found that meropenem pretreatment accelerates the mortality of mice subjected to severe AP. These data provide evidence that the mortality of AP mice is associated with meropenem-induced translocation of pathobionts such as *E. gallinarum*. These results reinforce and extend previous observations showing the potential harmful effects of antibiotics in inflammatory disease (3, 5).

Severe AP was induced by obstruction of the common biliopancreatic duct in mice, which is thought to be one of the best models resembling obstructive AP in patients. This model leads to severe AP characterized by necrosis of the pancreas and a high index of mortality (45). Pretreatment of AP mice with meropenem did not prevent infection, but instead induced MDR bacterial translocation into the blood. Our results diverge from previous studies demonstrating that meropenem prophylaxis reduces bacteremia, infection of the pancreatic tissue, and mortality of AP animals (24, 25). These studies administrated a single dose of meropenem in rats with AP induced by chemical agents. Several factors could account for these apparently conflicting results, but we speculate the microbiota differences between these species could be a key factor for the different results, since the antibiotic-induced emergence of MDR bacteria appears to be dependent of the animal source even in the same mice strain (3). Indeed, meropenem treatment-induced fast mortality of AP mice was observed in C57BL/6 and Balb/C mice house in our animal facility, but not in Swiss mice from a non-SPF animal facility where *E. gallinarum* is not prevalent in feces of these mice (data not shown). Our results are consistent with studies showing that pretreatment with broad-spectrum antibiotics induce dissemination of pathobionts by changing the microbiota composition (3–5). The mechanism involved in the spread of opportunistic bacteria from microbiota is poorly understood. However, antibiotic-decreased susceptible bacterial species could negatively impact the generation of antimicrobial peptides such as α-defensins, cryptdins, and the C-type lectin Reg3γ, which selectively kills Gram-positive bacteria (46–48).

Although *E. gallinarum* is a component of the mouse microbiota (49), only blood samples from meropenem-pretreated AP mice contained this bacterium. This evidence suggests that meropenem pretreatment favored bacterial growth following *E. gallinarum* dissemination and increased the mortality of AP mice. Accordingly, the infection of the AP mice or the microbiota enrichment with *E. gallinarum* recapitulated the accelerated mortality observed in the meropenem-pretreated AP mice. Pretreatment of AP mice with clindamycin and ceftriaxone, two antibiotics to which *E. gallinarum* showed resistance *in vitro*, also reproduced the meropenem effect on mortality. Because bacterial species other than *E. gallinarum* were identified on the blood of meropenem-pretreated AP mice, we cannot rule out the involvement of other bacteria species in the meropenem-accelerated mouse mortality. Nevertheless, our results indicate that *E. gallinarum* is sufficient to induce severe disease in AP mice.

*Enterococcus gallinarum* promoted lethality in naïve mice through a TLR2-dependent mechanism. This result is in agreement with previous reports, which demonstrated that the absence of a single TLR does not impair the local inflammatory response but reduces the systemic inflammatory response (30). Therefore, it is possible that other receptors/mechanisms, such as TLR6 (44), are involved in the recognition of *E. gallinarum* at the point of the infection. Furthermore, some studies have shown a key role for TLRs and their endogenous agonists in the severity of AP (50–54). For example, Sharif and colleagues (51) observed a significant reduction in the severity of AP in TLR4^−/−^ mice 24 h after caerulein or l-arginine administration. In accordance, Awla and colleagues (53) found that pancreatic damage, neutrophil infiltration, and CXCL2 in lung and pancreas were significantly reduced in TLR4^−/−^ mice, but not in TLR2^−/−^ mice, 24 h after AP induction by taurocholate infusion. On the other hand, Pastor and colleagues (54) observed a similar severity of AP in TLR4^−/−^ and Wt mice in the caerulein model. We did not observe a different mortality rate between TLR2^−/−^ and Wt mice in the obstructive model of AP (data not shown), suggesting that TLR2 does not play an important role in the final outcome of AP induced by biliopancreatitic duct obstruction. Nevertheless, the contribution of the gut microbiota diversity-TLRs axis in AP remains to be investigated.

Since the first report of bacteremia in humans by *E. gallinarum* (31), several VREs have been isolated from septic patients, and currently, they are a growing clinical problem (55). Infections by *E. gallinarum* have a low prevalence (1–3%) between VREs, but because most of the clinical samples have been reported as *Enterococcus* sp., the prevalence of *E. gallinarum* may be underestimated (56). Despite its low prevalence, *E. gallinarum* is invasive and can induce severe disease including bacteremia, endocarditis, meningitis, and peritonitis (57–59). In addition, *E. gallinarum* may represent a harmful role for the host in providing a large reservoir for resistance genes (60).

The role of microbiota in the severity of experimental AP is not fully understood, although bacterial translocation from the small intestine is a key event in this process (61). Together with the spread of *E. gallinarum* into the bloodstream, the meropenem pretreatment of AP mice also increased *E. gallinarum* in the small intestine. Bacteria culture-based methods used in the present work in bacteria identification are a less sensitive method when compared with culture-independent molecular approaches, such as 16S metagenomics (62, 63). Therefore, other bacteria species translocated into circulation or grew in low numbers in small intestine could be not detected by the conventional culture techniques. Even using less sensitive bacteria culture-based methods, a prevalent new pathobiont on the blood of meropenem-pretreated AP mice were identified. Accordingly, it has been demonstrated that antibiotic treatment of mice induces *Enterococcus* sp. colonization of the small intestine and a predominance of VREs in gut (4, 64). It might also be noted that *E. gallinarum* is an inhabitant of the small intestine in humans (65). In the context of pancreatic disease, a multi-hospital prospective clinical study showed that the intestinal population of *Enterococcus* is higher and more positively correlated with the serum levels of IL-6 in severe AP than in mild AP (66), suggesting that the increase in *Enterococcus* contributes to the severity of this disease. Despite *E. gallinarum* has not been associated with gallstone-induced obstructive AP in patients, a retrospective analysis of 56 cases of bacteremia caused by *E. casseliflavus* or *E. gallinarum* found these pathogens to be associated with biliary tract disease (67).

The present study provides evidence that the meropenem-induced fast mortality of AP mice is associated with the translocation of pathobionts, such as *E. gallinarum*. Although the data presented here using a murine model cannot be translated to clinical practice, our findings highlight the need for prospective clinical studies to evaluate the effect of meropenem on the MDR bacterial incidence in AP patients.

## Ethics Statement

Care and treatment of the animals were based on the Guide for the Care and Use of Laboratory Animals. All animal experiments were approved by the Animal Welfare Committee of the UFSC (PP00880).

## Author Contributions

FSS, FA, NS, LPS, LY, MS, FC, RF, LG, MV, and FS performed research, analyzed, and interpreted the data. JA-F and LPS contributed with analytic tools, data interpretation, and design of the work. AB and FS designed research, analyzed data, and wrote the paper. LKRS performed research, analyzed, and interpreted the data.

## Conflict of Interest Statement

The authors declare that the research was conducted in the absence of any commercial or financial relationships that could be construed as a potential conflict of interest.

## Abbreviations

AP, acute pancreatitis; BDO, biliopancreatic duct obstruction; MDR, multidrug-resistant; Sham, sham-operated; BW, body weight; CFU, colony-forming units; VRE, vancomycin-resistant enterococci.
