# Supplementary material for: Antibiotic-Induced Pathobiont Dissemination Accelerates Mortality in Severe Experimental Pancreatitis
Source: Front Immunol. 2017 Dec 22;8:1890. doi: 10.3389/fimmu.2017.01890 (PMC5770733; doi:10.3389/fimmu.2017.01890)
Supplement: Supplementary file 1 [file Data_Sheet_1.docx]

**Antibiotic-induced pathobiont dissemination accelerates mortality in severe experimental pancreatitis**

Fernanda S. Soares, Flávia C. Amaral, Natália L. C. Silva, Lorena K. R. Santos, Lívia H. Yamashiro, Mara C. Scheffer, Fernanda V. S. Castanheira, Raphael G. Ferreira, Laura Gehrke, Matheus R. Valente, José C. Alves-Filho, Luciano P. Silva, André Báfica, Fernando Spiller^*^.

^*^Correspondence: Fernando Spiller: [fernando.spiller@ufsc.br](mailto:fernando.spiller@ufsc.br)

**Supplemental Figures**

**
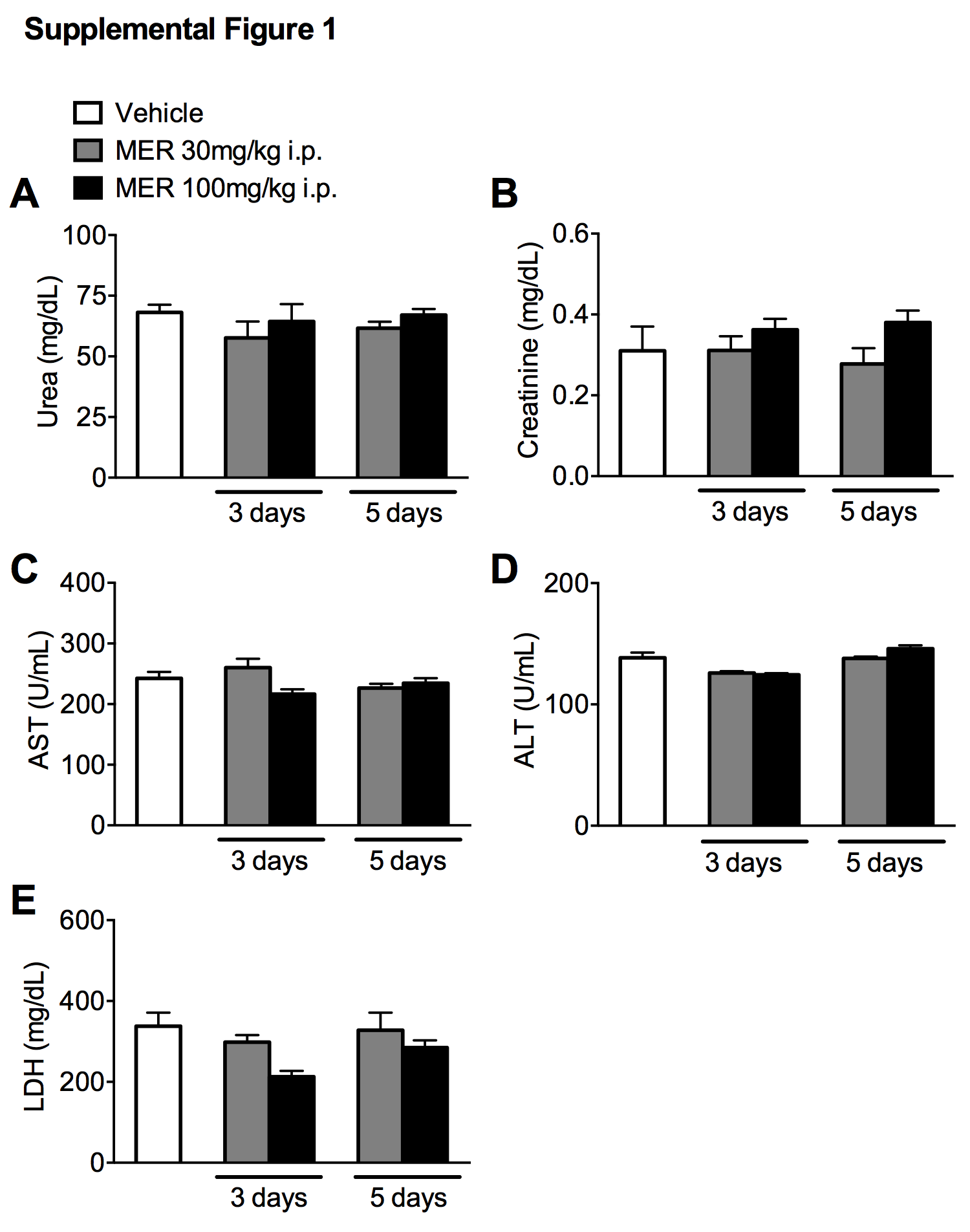
**

**Supplemental Figure 1. Treatment of naïve mice with meropenem does not induce toxicity.** Serum concentrations of (**A**) urea, (**B**) creatinine, (**C**) AST, (**D**) ALT and (**E**) LDH were evaluated 3 or 5 days after treatment with meropenem at 30 or 100 mg/kg, intraperitoneal (i.p.) 12/12 h for 3 (n = 10) or 5 days (n = 11). This experiment was performed independently two times. ALT = alanine aminotransferase; AST = aspartate aminotransferase; LDH = lactate dehydrogenase.


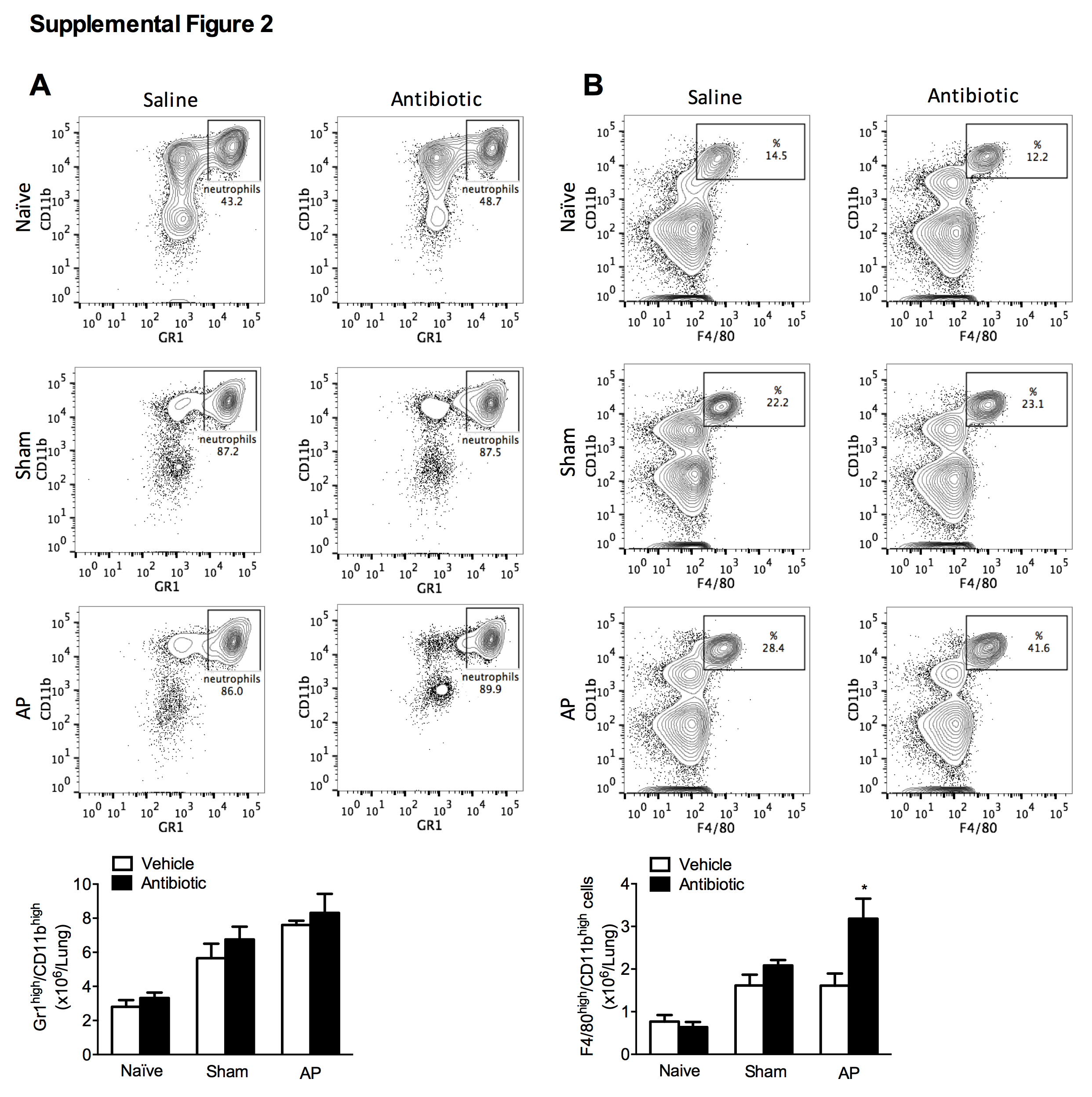


**Supplemental Figure 2. Leukocyte infiltration in lungs.** Enriched leukocytes suspensions from lungs were stained with anti-GR1, anti-CD11b and anti-F4/80. Doublets and autofluorescence cells were gated out from analysis. (**A**) Representative contour plots of CD11b vs GR1 showing the percentage of CD11b^high^GR1^high^ cells called “neutrophils”. (**B**) Contour plots of CD11b vs F4/80 showing the percentage of CD11b^high^F4/80^high^ cells called “monocytes”. Inserted in panel A and B are the absolute numbers of CD11b^high^GR1^high^ and CD11b^high^F4/80^high^ cells, respectively. Lungs were collected (16 h after the last dose meropenem) from naïve (n = 8), sham-operated (Sham, n = 8) and acute pancreatitis (AP, n = 10) mice i.p. pretreated with saline or meropenem (3 days, 12/12 h, 100 mg/kg, i.p.). Surgeries were performed 12 h after the last dose of antibiotic. This experiment was independently performed 2 times.


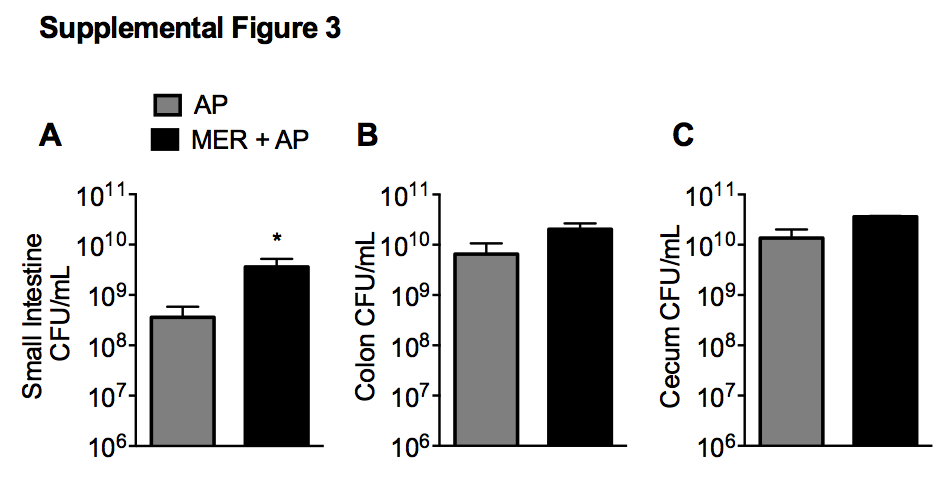


**Supplemental Figure 3. Increase in the CFU content in the small intestines of the meropenem-pretreated AP mice.** The CFU load was analyzed in the lumen of (**A**) the small intestine, (**B**) colon or (**C**) cecum 24 h after the surgeries. Mice were pretreated with saline (200 µL, i.p., 12/12 h, 3 days, n = 9) or meropenem (MER, 100 mg/kg, i.p., 12/12 h, 3 days, n = 12) and subjected to acute pancreatitis (AP). Surgeries were performed 12 h after the last treatment. **P<0.05* compared with the AP mice. This experiment was performed independently three times.


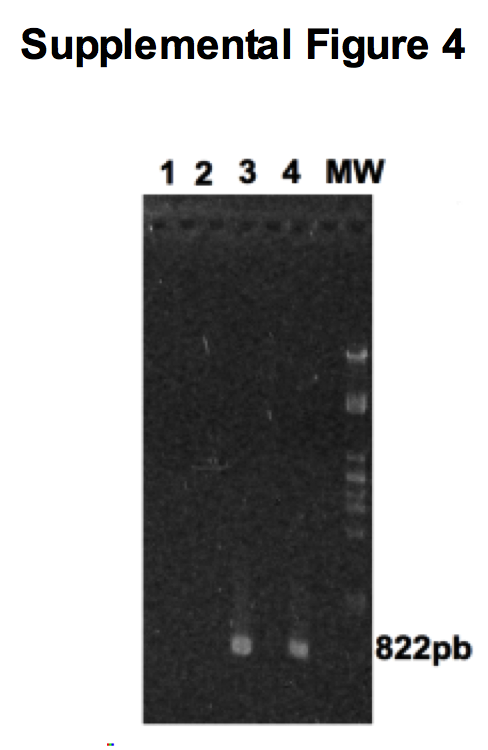


**Supplemental Figure 4. Molecular identification of the VanC1 gene in *Enterococcus gallinarum*.** The electrophoretic migration patterns of the amplified products by PCR using primers for the VanC1 gene. Lanes 1 and 2 show results from *Escherichia coli,* and lanes 3 and 4 show results from *E. gallinarum*. MW = size marker.


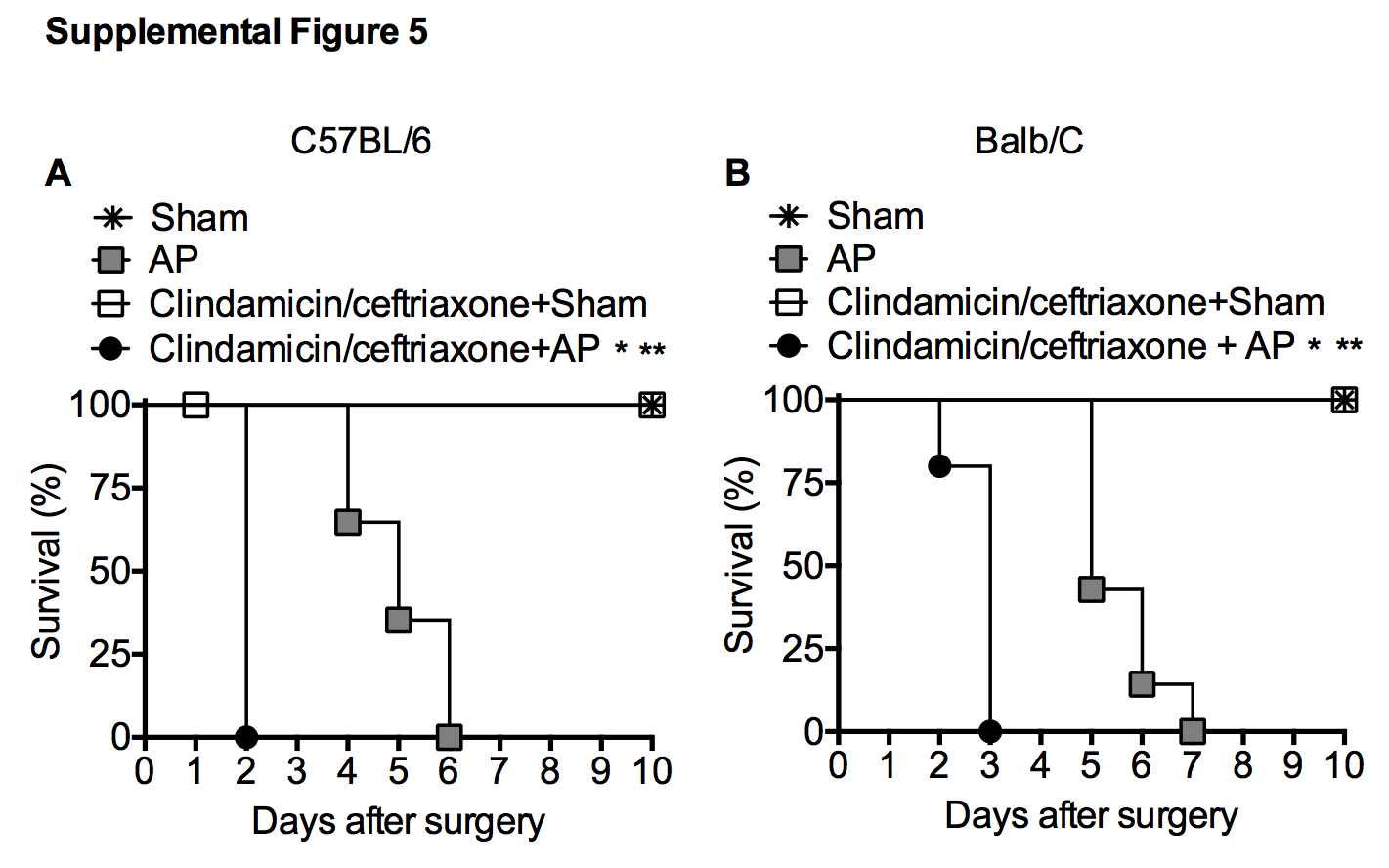


**Supplemental Figure 5. Pretreatment with clindamycin plus ceftriaxone accelerates the mortality rate of AP mice.** (**A**) Survival rate of C57BL/6 sham-operated (Sham, n = 8), acute pancreatitis (AP, n = 15) and acute pancreatitis mice pretreated i.p. 12/12 h for 3 days with clindamycin (25 mg/kg) plus ceftriaxone (30 mg/kg) (n = 15). The surgeries were performed 12 h after the last treatment. **P<0.0001* compared with the Sham mice. ***P<0.0001* compared with the AP mice. These experiments were performed independently three times. (**B**) Survival rate of Balb/C mice subjected to the same procedures in panel A. **P<0.0001* compared with the Sham mice. ***P<0.001* compared with the AP mice. These experiments were performed independently two times (Sham, n=8; AP n= 15).

**
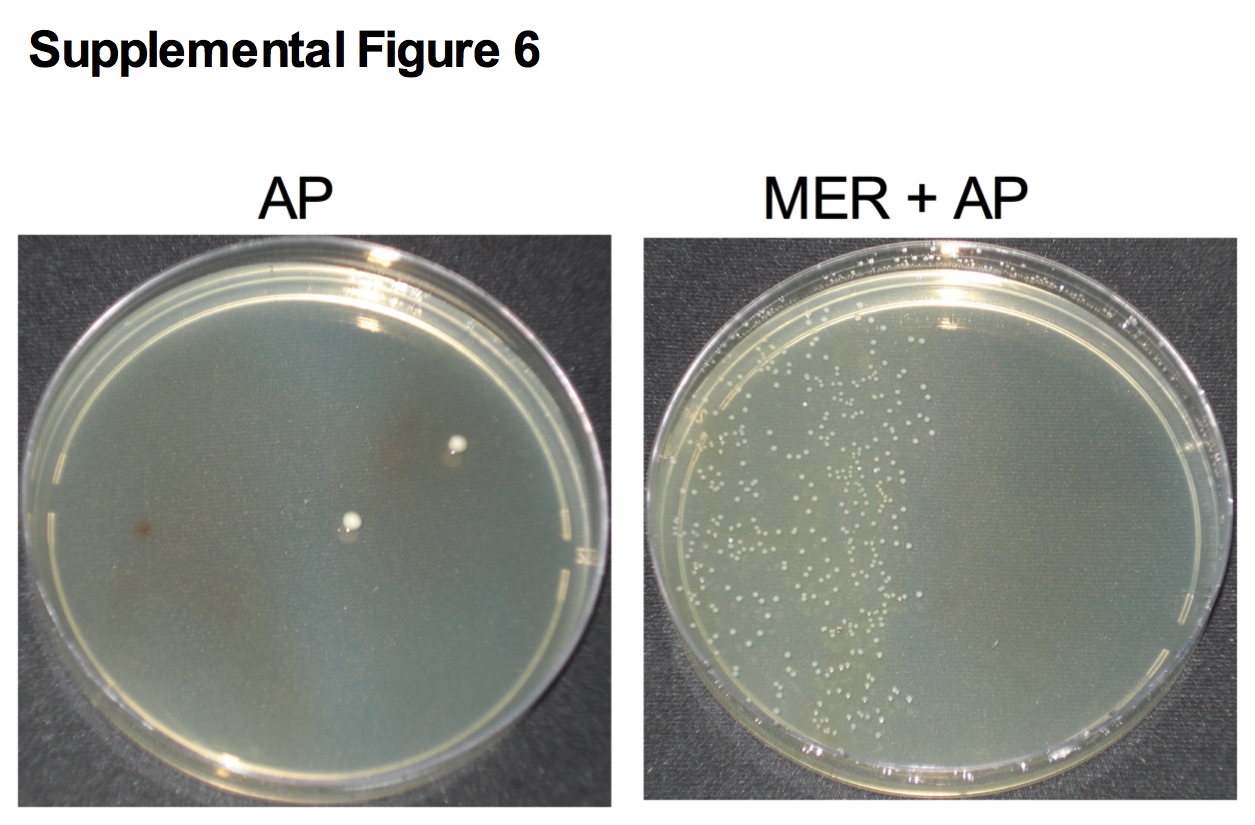
**

**Supplemental Figure 6.** Original pictures of the Mueller-Hinton agar plates from Figure 3C showing an example of the bacterial load in the blood samples from the acute pancreatitis (AP) or meropenem-pretreated AP (MER + AP) mice 24 h after the surgeries.

**Supplemental Tables**

Supplemental Table 1. Laboratory Report from Vitek2 showing the typical identification of *Enterococcus gallinarum*.

| Biochemical Details |
| --- |

| AMY | + | PIPLC | - | dXYL | + | ADH1 | + | BGAL | + | AGLU | - |
| --- | --- | --- | --- | --- | --- | --- | --- | --- | --- | --- | --- |
| APPA | - | CDEX | + | AspA | + | BGAR | + | AMAN | - | PHOS | - |
| LeuA | - | ProA | - | BGURr | - | AGAL | + | PyrA | + | BGUR | - |
| AlaA | + | TyrA | + | dSOR | - | URE | - | POLYB | + | dGAL | + |
| dRIB | + | ILATk | - | LAC | + | NAG | + | dMAL | + | BACI | + |
| NOVO | + | NC6.5 | + | dMAN | + | dMNE | + | MBdG | + | PUL | - |
| dRAF | + | O129R | + | SAL | + | SAC | + | dTRE | + | ADH2s | + |
| OPTO | + |  |  |  |  |  |  |  |  |  |  |

Card: GP (Gram-positive); Selected organism: *Enterococcus gallinarum* – 98% Probability. Confidence: Excellent identification. - = negative; + = positive; (-) or (+) = weak reactions close to the test threshold. ADH1 = arginine dihydrolase; ADH2S = arginine dihydrolase; AGAL = alpha-galactosidase; AGLU = alpha-glucosidase; AlaA = alanine arylamidase; AMAN = alpha-mannosidase; AMY = amygdalin; APPA = ala-phe-pro arylamidase; AspA = L-aspartic acid arylamidase; BACI = bacitracin resistance; BGAL = beta-galactosidase; BGAR = b-galactopurinosidase; BGUR = beta-glucorinidase; BGURr = beta-glucorinidase; CDEX = alpha-cyclodextrin; dGAL = galactose; dMAL = maltose; dMAN = mannitol; dMNE = mannose; dRAF = raffinose; dRIB = ribose; dSOR = sorbitol; dTRE = trehalose; dXYL = xylose; LAC = lactose; LeuA = L-leucine arylamidase; lLATK = lactate; MBDG = methyl-beta-D-glucopyranoside; NAG = *N*-acetyl-glucosamine; NC6.5 = growth in 6.5 NaCl; NOVO = novobiocin resistance; O129R = O/129 resistance; OPTO = optochin resistance; PHOS = alkaline phosphatase; PIPLC = phosphatidylinositol-Phospholipase C; POLYB = polymixin B resistance; ProA = Pro arylamidase beta-glucuronidase; PUL = pullulan; PYRA = L-pyroglutamic acid arylamidase; SAC = sucrose; SAL = salicin; TyrA = tyrosine arylamidase; URE = urease.

Table 2. Laboratory Report from Vitek2 showing the typical identification of *Staphylococcus epidermidis*.

| Biochemical Details |
| --- |

| AMY | - | PIPLC | - | dXYL | - | ADH1 | + | BGAL | - | AGLU | - |
| --- | --- | --- | --- | --- | --- | --- | --- | --- | --- | --- | --- |
| APPA | - | CDEX | - | AspA | - | BGAR | - | AMAN | - | PHOS | - |
| LeuA | - | ProA | - | BGURr | - | AGAL | - | PyrA | - | BGUR | - |
| AlaA | - | TyrA | - | dSOR | - | URE | + | POLYB | + | dGAL | + |
| dRIB | - | ILATk | + | LAC | + | NAG | - | dMAL | + | BACI | + |
| NOVO | - | NC6.5 | + | dMAN | - | dMNE | + | MBdG | - | PUL | - |
| dRAF | - | O129R | + | SAL | - | SAC | + | dTRE | - | ADH2s | - |
| OPTO | + |  |  |  |  |  |  |  |  |  |  |

Card: GP (Gram-positive); Selected organism: *Staphylococcus epidermidis* – 98% Probability. Confidence: Excellent identification. - = negative; + = positive; (-) or (+) = weak reactions close to the test threshold. ADH1 = arginine dihydrolase; ADH2S = arginine dihydrolase; AGAL = alpha-galactosidase; AGLU = alpha-glucosidase; AlaA = alanine arylamidase; AMAN = alpha-mannosidase; AMY = amygdalin; APPA = ala-phe-pro arylamidase; AspA = L-aspartic acid arylamidase; BACI = bacitracin resistance; BGAL = beta-galactosidase; BGAR = b-galactopurinosidase; BGUR = beta-glucorinidase; BGURr = beta-glucorinidase; CDEX = alpha-cyclodextrin; dGAL = galactose; dMAL = maltose; dMAN = mannitol; dMNE = mannose; dRAF = raffinose; dRIB = ribose; dSOR = sorbitol; dTRE = trehalose; dXYL = xylose; LAC = lactose; LeuA = L-leucine arylamidase; lLATK = lactate; MBDG = methyl-beta-D-glucopyranoside; NAG = *N*-acetyl-glucosamine; NC6.5 = growth in 6.5; NaCl; NOVO = novobiocin resistance; O129R = O/129 resistance; OPTO = optochin resistance; PHOS = alkaline phosphatase; PIPLC = phosphatidylinositol-Phospholipase C; POLYB = polymixin B resistance; ProA = Pro arylamidase beta-glucuronidase; PUL = pullulan; PYRA = L-pyroglutamic acid arylamidase; SAC = sucrose; SAL = salicin; TyrA = tyrosine arylamidase; URE = urease.

Supplemental Table 3. Laboratory Report from Vitek2 showing the typical identification of *Staphylococcus lentus*.

| Biochemical Details |
| --- |

| AMY | + | PIPLC | - | dXYL | + | ADH1 | (-) | BGAL | - | AGLU | - |
| --- | --- | --- | --- | --- | --- | --- | --- | --- | --- | --- | --- |
| APPA | - | CDEX | - | AspA | - | BGAR | - | AMAN | - | PHOS | - |
| LeuA | - | ProA | - | BGURr | - | AGAL | - | PyrA | + | BGUR | - |
| AlaA | - | TyrA | - | dSOR | + | URE | + | POLYB | - | dGAL | - |
| dRIB | + | ILATk | - | LAC | + | NAG | + | dMAL | + | BACI | + |
| NOVO | + | NC6.5 | + | dMAN | + | dMNE | + | MBdG | + | PUL | - |
| dRAF | + | O129R | + | SAL | + | SAC | + | dTRE | + | ADH2s | - |
| OPTO | + |  |  |  |  |  |  |  |  |  |  |

Card: GP (Gram-positive); Selected organism: *Staphylococcus lentus* – 96% Probability. Confidence: Excellent identification. - = negative; + = positive; (-) or (+) = weak reactions close to the test threshold. ADH1 = arginine dihydrolase; ADH2S = arginine dihydrolase; AGAL = alpha-galactosidase; AGLU = alpha-glucosidase; AlaA = alanine arylamidase; AMAN = alpha-mannosidase; AMY = amygdalin; APPA = ala-phe-pro arylamidase; AspA = L-aspartic acid arylamidase; BACI = bacitracin resistance; BGAL = beta-galactosidase; BGAR = b-galactopurinosidase; BGUR = beta-glucorinidase; BGURr = beta-glucorinidase; CDEX = alpha-cyclodextrin; dGAL = galactose; dMAL = maltose; dMAN = mannitol; dMNE = mannose; dRAF = raffinose; dRIB = ribose; dSOR = sorbitol; dTRE = trehalose; dXYL = xylose; LAC = lactose; LeuA = L-leucine arylamidase; lLATK = lactate; MBDG = methyl-beta-D-glucopyranoside; NAG = *N*-acetyl-glucosamine; NC6.5 = growth in 6.5; NaCl; NOVO = novobiocin resistance; O129R = O/129 resistance; OPTO = optochin resistance; PHOS = alkaline phosphatase; PIPLC = phosphatidylinositol-Phospholipase C; POLYB = polymixin B resistance; ProA = Pro arylamidase beta-glucuronidase; PUL = pullulan; PYRA = L-pyroglutamic acid arylamidase; SAC = sucrose; SAL = salicin; TyrA = tyrosine arylamidase; URE = urease.

Supplemental Table 4. Laboratory Report from Vitek2 showing the typical identification of *Enterobacter clocae complex*.

| Biochemical Details |
| --- |

| APPA | - | ADO | - | PyrA | - | IARL | - | dCEL | + | BGAL | + |
| --- | --- | --- | --- | --- | --- | --- | --- | --- | --- | --- | --- |
| H2S | - | BNAG | + | AGLTp | - | dGLU | + | GGT | + | OFF | + |
| BGLU | + | dMAL | + | dMAN | + | dMNE | + | BXYL | + | BAlap | - |
| ProA | + | LIP | - | PLE | + | TyrA | + | URE | - | dSOR | + |
| SAC | + | dTAG | - | dTRE | + | CIT | + | MNT | - | 5KG | - |
| ILATk | + | AGLU | - | SUCT | + | NAGA | + | AGAL | + | PHOS | - |
| GlyA | + | ODC | + | LDC | - | IHISa | - | CMT | - | BGUR | - |
| O129R | + | GGAA | - | IMLTa | - | ELLM | - | ILATa | - |  |  |

Card: GN (Gram-negative); Selected organism: *Enterobacter clocae complex* – 95% Probability. Confidence: Very good identification. (-) Negative; (+) positive. (-) or (+) = weak reactions close to the test threshold. 0DEC = Decarboxylase base; 5KG = 5-Keto-D-gluconate; ADO = Adonitol; AGAL = Alpha-galactosidase; AGLTp = Glutamyl Arylamidase pNA; AGLU = Alpha-glucosidase; APPA = Ala-Phe-arylamidase; BAlap = Beta-alanine arylamidase pNA; BGAL = Beta-galactosidase; BGLU = Beta-glucosidase; BGUR = Beta-glucoronidase; BNAG = Beta-N-acetyl-glucosaminidase; BXYL = Beta-xylosidase; CIT = Citrate (Sodium); CMT = Coumarate; dCEL = D-cellobiose; dGLU = D-glucose; dMAL = D-maltose; dMAN = D-manitol; dMNE = D-mannose; dSOR = D-sorbitol; dTAG = D-tagatose; dTRE = D-trelose; ELLM = Ellman; GGAA = Glu-Gly-Arg-arylamidase; GGT = Gamma-glutamyl-transferase; GlyA = Glycine arylamidase; H2S = H2S production; IARL = L-arabitol; IHISa = L-histidine assimilation; ILATa = L-lactate assimilation; ILATk = L-lactate alkalinisation; IMLTa = L-malate assimilation; LDC = Lysine decarboxylase; LIP = Lipase; MNT = Malonate; NAGA = Beta-N-acetyl-galactosaminidase; O129R = O/129 Resistance (Comp.vibrio.); ODC = Ornithine decarboxylase; OFF = Fermentation/Glucose; PHOS = Phosphatase; PLE = Palatinose

ProA = L-proline arylamidase; PyrA = L-pyrrolydonyl-arylamidase; SAC = Saccharose/Sucrose; SUCT = Succinate alkalinisation; TryA = Tyrosine arylamidase;

URE = Urease.

Supplemental Table 5. Laboratory Report from Vitek2 showing the typical identification of *Staphylococcus warneri*.

| Biochemical Details |
| --- |

| AMY | - | PIPLC | - | dXYL | - | ADH1 | + | BGAL | - | AGLU | - |
| --- | --- | --- | --- | --- | --- | --- | --- | --- | --- | --- | --- |
| APPA | - | CDEX | - | AspA | - | BGAR | - | AMAN | - | PHOS | - |
| LeuA | - | ProA | - | BGURr | - | AGAL | - | PyrA | - | BGUR | + |
| AlaA | - | TyrA | - | dSOR | - | URE | + | POLYB | - | dGAL | - |
| dRIB | (-) | ILATk | + | LAC | - | NAG | - | dMAL | + | BACI | + |
| NOVO | - | NC6.5 | + | dMAN | - | dMNE | - | MBdG | - | PUL | - |
| dRAF | - | O129R | + | SAL | - | SAC | + | dTRE | + | ADH2s | - |
| OPTO | + |  |  |  |  |  |  |  |  |  |  |

Card: GP (Gram-positive); Selected organism: *Staphylococcus warneri* – 97% Probability. Confidence: Excellent identification. - = negative; + = positive; (-) or (+) = weak reactions close to the test threshold. ADH1 = arginine dihydrolase; ADH2S = arginine dihydrolase; AGAL = alpha-galactosidase; AGLU = alpha-glucosidase; AlaA = alanine arylamidase; AMAN = alpha-mannosidase; AMY = amygdalin; APPA = ala-phe-pro arylamidase; AspA = L-aspartic acid arylamidase; BACI = bacitracin resistance; BGAL = beta-galactosidase; BGAR = b-galactopurinosidase; BGUR = beta-glucorinidase; BGURr = beta-glucorinidase; CDEX = alpha-cyclodextrin; dGAL = galactose; dMAL = maltose; dMAN = mannitol; dMNE = mannose; dRAF = raffinose; dRIB = ribose; dSOR = sorbitol; dTRE = trehalose; dXYL = xylose; LAC = lactose; LeuA = L-leucine arylamidase; lLATK = lactate; MBDG = methyl-beta-D-glucopyranoside; NAG = *N*-acetyl-glucosamine; NC6.5 = growth in 6.5; NaCl; NOVO = novobiocin resistance; O129R = O/129 resistance; OPTO = optochin resistance; PHOS = alkaline phosphatase; PIPLC = phosphatidylinositol-Phospholipase C; POLYB = polymixin B resistance; ProA = Pro arylamidase beta-glucuronidase; PUL = pullulan; PYRA = L-pyroglutamic acid arylamidase; SAC = sucrose; SAL = salicin; TyrA = tyrosine arylamidase; URE = urease.

Supplemental Table 6. Laboratory Report from Vitek2 showing the typical identification of *Raoultella planticola*.

| Biochemical Details |
| --- |

| APPA | - | ADO | + | PyrA | + | IARL | - | dCEL | + | BGAL | + |
| --- | --- | --- | --- | --- | --- | --- | --- | --- | --- | --- | --- |
| H2S | - | BNAG | + | AGLTp | - | dGLU | + | GGT | - | OFF | + |
| BGLU | + | dMAL | + | dMAN | + | dMNE | + | BXYL | + | BAlap | - |
| ProA | - | LIP | - | PLE | + | TyrA | + | URE | + | dSOR | + |
| SAC | + | dTAG | - | dTRE | + | CIT | + | MNT | + | 5KG | + |
| ILATk | + | AGLU | - | SUCT | + | NAGA | + | AGAL | + | PHOS | + |
| GlyA | + | ODC | - | LDC | + | IHISa | - | CMT | - | BGUR | - |
| O129R | + | GGAA | - | IMLTa | - | ELLM | + | ILATa | - |  |  |

Card: GN (Gram-negative); Selected organism: *Raoultella planticola* – 99% Probability. Confidence: Excellent identification. - = Negative; + = positive. 0DEC = Decarboxylase base; 5KG = 5-Keto-D-gluconate; ADO = Adonitol; AGAL = Alpha-galactosidase; AGLTp = Glutamyl Arylamidase pNA; AGLU = Alpha-glucosidase; APPA = Ala-Phe-arylamidase; BAlap = Beta-alanine arylamidase pNA; BGAL = Beta-galactosidase; BGLU = Beta-glucosidase; BGUR = Beta-glucoronidase; BNAG = Beta-N-acetyl-glucosaminidase; BXYL = Beta-xylosidase; CIT = Citrate (Sodium); CMT = Coumarate; dCEL = D-cellobiose; dGLU = D-glucose; dMAL = D-maltose; dMAN = D-manitol; dMNE = D-mannose; dSOR = D-sorbitol; dTAG = D-tagatose; dTRE = D-trelose; ELLM = Ellman; GGAA = Glu-Gly-Arg-arylamidase; GGT = Gamma-glutamyl-transferase; GlyA = Glycine arylamidase; H2S = H2S production; IARL = L-arabitol; IHISa = L-histidine assimilation; ILATa = L-lactate assimilation; ILATk = L-lactate alkalinisation; IMLTa = L-malate assimilation; LDC = Lysine decarboxylase; LIP = Lipase; MNT = Malonate; NAGA = Beta-N-acetyl-galactosaminidase; O129R = O/129 Resistance (Comp.vibrio.); ODC = Ornithine decarboxylase; OFF = Fermentation/Glucose; PHOS = Phosphatase; PLE = Palatinose

ProA = L-proline arylamidase; PyrA = L-pyrrolydonyl-arylamidase; SAC = Saccharose/Sucrose; SUCT = Succinate alkalinisation; TryA = Tyrosine arylamidase;

URE = Urease.

Supplemental Table 7. Standard microbiological and biochemical tests used for *Enterococcus gallinarum* identification.

| Biochemical Details and antibiotic resistance profile |
| --- |

| Gram staining  Catalase production  Growth in 6.5% NaCl  Esculin hydrolysis  Clindamycin | Gram-positive cocci  -  +  +  R | Ceftriaxone  Streptomycin  Meropenem  Vancomycin  Ampicillin | R  R  I  I  S |
| --- | --- | --- | --- |

- = negative; + = positive; I = intermediate; R = resistant; S = susceptible. Antibiotic resistance profile was analyzed by disk diffusion method.

Supplemental Table 8. Standard microbiological and biochemical tests used for *Bacillus* spp. identification.

| Biochemical Details |
| --- |

| Gram staining  Catalase production | Gram-positive rods  + | Oxidase test  Motility | +  + |
| --- | --- | --- | --- |

- = negative; + = positive.
